# Animal Perception of Seasonal Thresholds: Changes in Elephant Movement in Relation to Rainfall Patterns

**DOI:** 10.1371/journal.pone.0038363

**Published:** 2012-06-27

**Authors:** Patricia J. Birkett, Abi T. Vanak, Vito M. R. Muggeo, Salamon M. Ferreira, Rob Slotow

**Affiliations:** 1 Amarula Elephant Research Programme, School of Life Sciences, University of Kwazulu-Natal, Durban, South Africa; 2 Dipartimento di Scienze Statistiche e Matematiche ‘Vianelli’, Università di Palermo, Palermo, Italy; 3 Scientific Services, Kruger National Park, Skukuza, South Africa; University of California, Berkeley, United States of America

## Abstract

**Background:**

The identification of temporal thresholds or shifts in animal movement informs ecologists of changes in an animal’s behaviour, which contributes to an understanding of species’ responses in different environments. In African savannas, rainfall, temperature and primary productivity influence the movements of large herbivores and drive changes at different scales. Here, we developed a novel approach to define seasonal shifts in movement behaviour by examining the movements of a highly mobile herbivore (elephant; *Loxodonta africana*), in relation to local and regional rainfall patterns.

**Methodology/Principal Findings:**

We used speed to determine movement changes of between 8 and 14 GPS-collared elephant cows, grouped into five spatial clusters, in Kruger National Park, South Africa. To detect broad-scale patterns of movement, we ran a three-year daily time-series model for each individual (2007–2009). Piecewise regression models provided the best fit for elephant movement, which exhibited a segmented, waveform pattern over time. Major breakpoints in speed occurred at the end of the dry and wet seasons of each year. During the dry season, female elephant are constrained by limited forage and thus the distances they cover are shorter and less variable. Despite the inter-annual variability of rainfall, speed breakpoints were strongly correlated with both local and regional rainfall breakpoints across all three years. Thus, at a multi-year scale, rainfall patterns significantly affect the movements of elephant. The variability of both speed and rainfall breakpoints across different years highlights the need for an objective definition of seasonal boundaries.

**Conclusions/Significance:**

By using objective criteria to determine behavioural shifts, we identified a biologically meaningful indicator of major changes in animal behaviour in different years. We recommend the use of such criteria, from an animal’s perspective, for delineating seasons or other extrinsic shifts in ecological studies, rather than arbitrarily fixed definitions based on convention or common practice.

## Introduction

The study of animal movement patterns allows ecologists to determine the distribution of species both in space and time, and the factors that influence their movements in different environments [1]. Spatial variation in the landscape results in a heterogeneous distribution of resources such as habitats, water, and forage patches [2], [3]. However, the time-frames over which these resources are available to an individual also vary, and are influenced by abiotic factors such as rainfall and temperature [4]. For example, in African savanna systems, forage resources may vary according to seasonal changes in rainfall [5] and animals respond to these conditions by altering or shifting their patterns of movement over time [6]. Such variations in responses impose a range of challenges when conservationists seek specific outcomes. Decision makers can thus be better informed by defining appropriate temporal scales over which shifts in species movement patterns occur.

Recent advances in animal-mounted GPS technology has increased the availability of fine-scale animal movement data, thus enhancing our ability to better understand patterns in animal movement behaviour [7], [8]. Several studies have investigated the fine-scale ranging behaviour of large mammals, including elk (*Cervus elaphus*) [6], moose (*Alces alces*) [9], caribou (*Rangifer tarandus*) [2] and African elephant (*Loxodonta africana*) [10], [11]. Many of these studies focussed on defining the movement ‘modes’ or types of movement of animals across seasons, and over various scales of resolution. In each case, seasons were designated based on climatic proxies such as temperature, rainfall or snowfall. Although these variables may have a direct influence on the movement behaviour of the animal, this method imposes seasonal boundaries which may not necessarily reflect the natural variation in movement patterns of the animal within the ecosystem, i.e. the responses of the study animal itself. Furthermore, ecological changes, including changes in both abiotic and biotic factors across temporal boundaries such as seasons, often impose a constraint or release on an animal’s behaviour [12]. We suggest that a process which allows the movement patterns of an animal to define these temporal boundaries may be more informative (e.g. [13]).

Large herbivores are highly mobile and able to cover large distances across the landscape. Since they encounter forage resources at various levels of spatial and temporal scale [5], it is important to consider the effect of scale in studies of these animals (see [14]). Patterns of herbivore movement over time also vary in scale; for example, fine-scale patterns may include short periods of foraging, searching for food, and resting, while extended periods of exploration may span more than a day [15], [16]. At broader scales; monthly, seasonal, annual and even inter-annual patterns of movements may be detected [6], [15].

In this study, we focus on defining broad-scale temporal changes in the movement behaviour of female elephant. The African elephant is considered to be a keystone species in African savanna systems, since elephant foraging behaviour affects various ecosystem processes [17]. Although there is extensive literature on the seasonal space use or home range dynamics of elephant from many areas in southern Africa [18]–[25], these studies have pre-defined ‘wet’ and ‘dry’ periods, usually inferred from regional rainfall records, and arbitrarily designated according to calendar months (e.g.: ‘wet’ period: October – March). Here, we apply a different approach by considering animal perception of seasonal change: we examine movement behaviour in order to identify ecologically significant thresholds (i.e. breakpoints) over time. Thus, we aim to define an unbiased temporal scale over which changes in the movement patterns of elephant can be detected. We then examine whether these shifts in elephant movement can be related to rainfall patterns at local and regional scales.

Because of the broad-scale effects of rainfall on both water availability and vegetation phenology and biomass [4], we hypothesised that, in general, changes in elephant movements over time would be affected by rainfall patterns [25]. We expected major breakpoints in elephant movement to occur with the onset of the first rains of the season in each year. We also predicted that elephant would be more strongly affected by local rather than broader regional rainfall patterns across all years. We expected additional minor breakpoints to occur at other points during the year, in which elephant may be responding to factors other than rainfall. However, our focus was to define the major seasonal breakpoints, in particular, the breakpoint at the end of the dry season. During this period, elephant will most likely respond to an increase in rainfall, coupled with an increase in forage quality and biomass, resulting in a release from constrained movement behaviour, typical of late dry-season conditions [12].

## Materials and Methods

### Ethics Statement

Elephant capture & handling was conducted in strict accordance with ethical standards. Specific approval for this particular research project was obtained through the University of KwaZulu-Natal Animal Ethics sub-committee (Ref. 009/10/Animal). This research also forms part of a registered and approved SANParks project, in association with Kruger National Park and Scientific Services (Ref: BIRPJ743).

**Figure 1:**
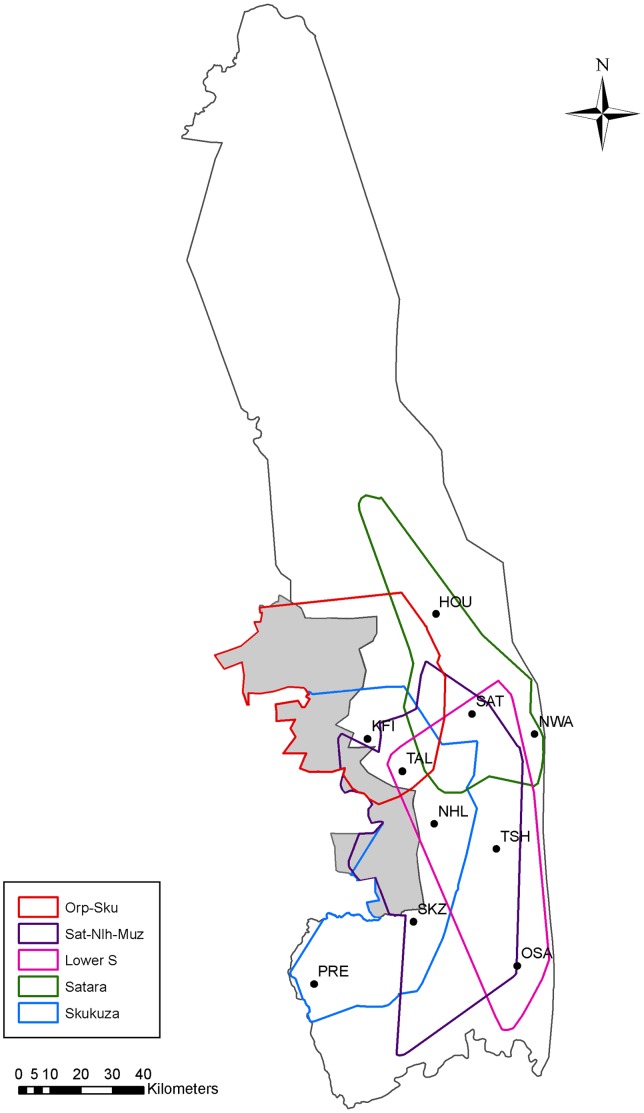
The distribution of elephant clusters in The Kruger National Park and associated private reserves. The KNP boundary is outlined in grey, with the contiguous private reserves shaded in grey. The five clusters are shown as minimum convex polygons (MCP’s), which have been clipped according to the boundaries of Kruger and other Private Reserves. These clusters are based on the distribution of collared female elephant over the entire study period. MCP’s were calculated for three collars in the Orpen-Skukuza cluster, four collars in the Satara-Nhlanguleni-Muzanduzi cluster, one collar in the Lower Sabie cluster, four collars in the Satara cluster and three collars in the Skukuza cluster. The 10 rainfall stations used are abbreviated: HOU– Houtboschrand, SAT – Satara, NWA – Nwanetsi, TSH – Tshokwane, OSA – Lower Sabie, SKZ – Skukuza, PRE – Pretoriuskop, NHL – Nlhanguleni, TAL – Talamati, KFT – Kingfisherspruit.

The Kruger National Park (KNP) and associated private reserves along the western boundary (Sabie Sand, Klaserie, Timbavati, Umbabat and Manyaleti), extend across an area of approximately 21,281 km^2^, in the north-eastern Lowveld region of the South Africa. Our study area covers the southern, central and western regions of KNP and includes the associated private reserves, since elephant are able to move freely between these areas (Fig. 1). Vegetation in this region is primarily classified as semi-arid to arid wooded savanna [26].

The elephant population in KNP was estimated to be ∼ 14,000 individuals during 2010 (SANParks, unpublished data). From 2006 to 2010, we collected geographical location data, downloaded from GPS/GSM Collars (Africa Wildlife Tracking cc., South Africa), fitted to 17 elephant cows from different herds. To ensure the independence of sampling, a single female in each herd was selected and collared. The movements of these collared females are thus assumed to represent the movement behaviour of the breeding herd to which they belong [27], [28]. The females were categorised according to five ‘clusters’, based on the area in which they were collared: Orpen-Skukuza, Satara-Nhlanguleni-Muzanduzi, Lower Sabie, Satara and Skukuza (Fig. 1). The Lower Sabie cluster included four collars, however three of these malfunctioned. Thus we were only able to use a single collar for this cluster and 14 collars in total. All herds were distributed between −25.37°S in the south and −23.75°S in the north; 32.00°E in the east and 30.99°E in the west. To maintain data capture at a relatively high temporal resolution, the collars were set to record locations at 30 min intervals. We obtained PDOP (Positional dilution of precision) values from six of the collars. PDOP is a 3-D measure of the quality of GPS data, where lower values usually indicate higher location accuracy [29]. The average value obtained was 1.82 with a variance of 0.76, indicating a low error in position estimation. We assumed other collars to have similar levels of error.

To examine the temporal scale over which elephant movement behaviour changed, we calculated the mean daily speed (km/h) and the variance (standard deviation) associated with the speed. To calculate these variables, we computed step-lengths at 30 min intervals using Hawth’s Tools [30] in ArcGIS 9.3 (Environmental Systems Research Institute, Redlands, CA, USA) and then converted this to speed (km/hr). To minimise the effect of acquisition errors, we used only those step-length values recorded within the interval 27.5–32.5 min. We checked for errors where step-lengths appeared to be either unusually long or abnormal, by converting points to paths using Hawth’s tools [30]. Cases of obvious errors were either corrected where possible, or removed from the dataset. Because of data errors, we were not able to use 91 days of data across all collars in all years (2007: average 1.9 days per collar; 2008∶1.8 days per collar: 2009∶6.1 days per collar). We were able to use full-year datasets from eight collars in 2007, 14 collars in 2008 and eight collars in 2009.

We analysed the daily time series for each elephant separately using a piece-wise linear or segmented regression model (hereafter PRM), since this provides a useful method for determining ecological thresholds [31]. We estimated breakpoints via the algorithm described in [32] and implemented in R 2.12 [33] using the package ‘segmented’, version 0.2–7.2 [34]. Due to the positive support of the response variable and the observed ‘waveform’ temporal patterns, we assumed a generalised linear model (hereafter GLM) with a log-link function (Poisson distribution) and an identity variance function, with multiple breakpoints. Namely, for each elephant:
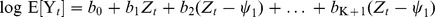
where *Y_t_* is the random variable representing the elephant movement at day *t*, *E[Y_t_]* is its expected value to be expressed as a segmented function of day with parameters *ψ* and *b*. More specifically, *ψ* represents the breakpoints, i.e. the days where the elephant movement changes, and *b* regulates the slopes in the different time periods. *Z_t_* is the generic numeric covariate representing the time index, which has a piecewise linear relationship with the response variable, *Y_t_.*

We ensured that breakpoints were valid by checking the corresponding gap coefficient and its *t*-value, (breakpoint accepted when *t*<2, [35]). In order to select the most appropriate model, we compared Bayesian Information Criteria (hereafter BIC) values (see [32], [36]) for the two models (GLM, PRM). We also calculated the bootstrapped 95% confidence intervals for the breakpoints in the PRM’s.

To calculate a measure of rainfall, we applied two methods. For a regional measure of rainfall, we used rainfall values averaged from ten stations (see Fig. 1) within the combined spatial area used by all herds. To obtain rainfall values at a finer scale, we averaged values from between one and three stations located within the spatial range of individual herds. These represented ‘local’ rainfall for individual herds in separate years. We chose local stations by visually inspecting individual location points in relation to the position of a station.

Following the methods used to model step length variables, we fitted GLM’s and PRM’s to the rainfall data and obtained breakpoints by using the algorithm described in [32]. We then compared BIC values to determine the best fit for the rainfall models.

We ran 1-tailed bivariate correlations to identify the relationship between local rainfall and elephant movement, and regional rainfall and elephant movement. To check whether ‘year and ‘cluster’ were confounding variables, we also ran 1-tailed partial correlations using: all breakpoints, and then upper and lower breakpoints separately. In each case, we controlled for the variables together, and then separately. Statistical analyses were conducted in PASW Statistics 18, (SPSS Inc., 2009, Chicago IL).

## Results

The analysis of elephant movement patterns revealed that in all cases, the Piecewise Regression Model (PRM) provided a better fit than the Generalised Linear Model (GLM). The movement patterns of female elephant exhibited a distinct waveform trend over the three-year period, with behavioural changes occurring at the transition between both the wet and dry season, and the dry and wet season. Thus we obtained dry-wet season breakpoints at the troughs within the model, (hereafter referred to as ‘lower’ speed breakpoints) and wet-dry season breakpoints at the peaks (hereafter referred to as ‘upper’ speed breakpoints) (see Appendix S1). Although we detected 2 additional breakpoints for collar AM99 in 2008, and an additional breakpoint for AM108 in 2008, these were considered minor breakpoints (based on BIC values), and were removed from the correlation analysis. The major breakpoints were of primary interest, since the aim of the study was to define broad-scale shifts in elephant movement over time.

**Figure 2:**
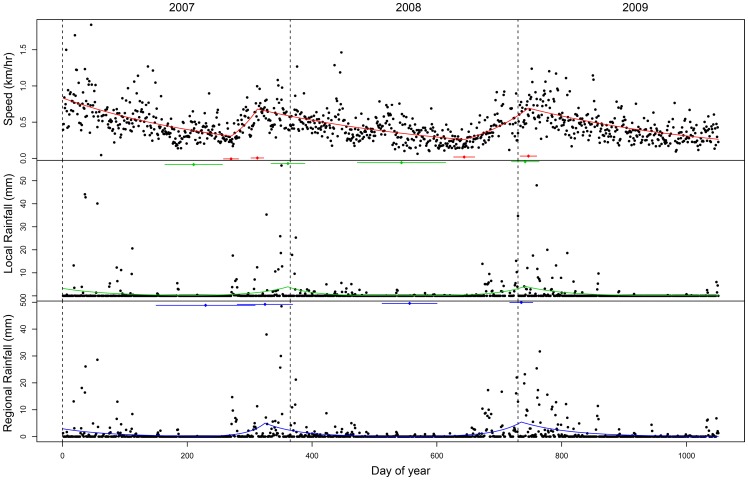
Multiyear piecewise regression models for Collar AM93, within the period 2007–2009, in Kruger National Park. Variables modelled include: average speed in the upper row (red line), average local rainfall in the middle row (green line), and average regional rainfall in the lower row (blue line). The columns (separated by dashed-lines) represent different years: 2007, 2008 and 2009 from left to right. The X axis represents ‘day of year’. Breakpoints are given (in associated colours), together with the 95% confidence interval for each breakpoint (represented by horizontal bars).

In all years, the relationship between both speed and variance of speed, and day of year was negative during the period between the wet and dry season, followed by a breakpoint at the dry-wet season boundary. Beyond this, as the wet season commenced, the relationship was positive, up until a breakpoint at the end of the wet season (example AM93, Fig. 2). In one case, (AM108 in 2008), an initial lower breakpoint occurred at approximately day 50 (mid-February) in 2008. Between the 14^th^ of June and the 7^th^ of July, this elephant increased her average speed on 11 of the 23 days (>0.5km/h). Examination of AM108’s movement pattern during this period indicated that she was in close association with AM106 within the south of Kruger from the 15^th^ until the 19^th^ of June, where after she began moving rapidly and directly, across Sabi Sand Reserve, and into the central region of Kruger, covering a distance of approx. 50 km in 3 days (19^th^–21^st^ June). From this point, she moved further into the northern section of her range. AM108’s increased movements may be the result of a disturbance event [37], although it is more likely that she is moving in order to access forage, possibly as a result of limited resources in the southern areas of Kruger during this dry period, or because of competitive exclusion by AM106 (e.g. see [10]). AM108’s speed breakpoint at day 50 occurred because of the increase in average speed over this period in June-July. As a result of this ‘peak’ in speed, the breakpoint at 551 weeks is an ‘upper’ breakpoint, when it would usually be a ‘lower’ breakpoint. Thus for AM108 in 2008, the shape of the curve, and by implication, the movement pattern, appears inverted. We have removed this outlier ‘upper’ breakpoint, along with corresponding local and regional rainfall breakpoints for AM108 in 2008, from the correlation analysis.

Individual collar breakpoints in speed varied between years, with lower breakpoints occurring, on average, on day 249 (approx. 6^th^ September) in 2007 (95% CI ∼ 128 days), day 614 (approx. 5^th^ September) in 2008 (95% CI ∼ 160 days), and day 950 (approx. 7^th^ August) in 2009 (%95 CI ∼ 138 days) (Appendix S2). Upper breakpoints occurred, on average, on day 320 (approx. 16^th^ November) in 2007 (95% CI ∼ 41 days). There were only 2 breakpoints in 2008, for collars AM306 and AM307) (day 717; approx. 17^th^ December) (95% CI ∼ 1 day). In 2009, breakpoints occurred on day 754 (approx. 23rd January) (95% CI ∼ 16 days) (Appendix S2).

For rainfall patterns, the PRM’s provided a better fit, and the models also identified a distinct waveform relationship, between both local and regional rainfall, and day of year. Thus, as with speed breakpoints, we also obtained dry-wet season breakpoints at the troughs within the local and regional rainfall models, (hereafter referred to as ‘lower’ rainfall breakpoints) and wet-dry season breakpoints at the peaks (hereafter referred to as ‘upper’ rainfall breakpoints) (see Appendix S1). Major local rainfall breakpoints varied between years, with lower breakpoints occurring, on average, on day 225 (approx. 13^th^ August) in 2007 (95% CI ∼ 93 days), day 543 (approx. 26^th^ June) in 2008 (95% CI ∼ 115 days), and day 956 (approx.13^th^ August) in 2009 (95% CI ∼ 121 days) (Appendix S2). Local rainfall (upper) breakpoints occurred on average, on day 343 (approx. 9^th^ December) in 2007 (95% CI ∼ 90 days). There was only one breakpoint in 2008, for the collar AM306 (day 696; approx. 26^th^ November). In 2009, breakpoints occurred on day 764 (approx. 2^nd^ February) (95% CI ∼ 41 days) (see Appendix S2).

Major regional rainfall breakpoints (lower) occurred, on average, on day 222 (approx. 10^th^ August) in 2007 (95% CI ∼ 102 days); day 561 (approx. 14^th^ July) in 2008 (95% CI ∼ 43 days); and day 962 (approx. 19^th^ August) in 2009 (95% CI ∼ 60 days), (Appendix S2). Major regional rainfall breakpoints (upper) occurred, on average, on day 330 (approx. 26^th^ November) in 2007 (95% CI ∼ 83 days), no upper breakpoints for regional rainfall occurred in 2008; and in 2009, breakpoints occurred on day 742 (approx. 11^th^ January) (95% CI ∼ 8 days) (Appendix S2).

**Figure 3:**
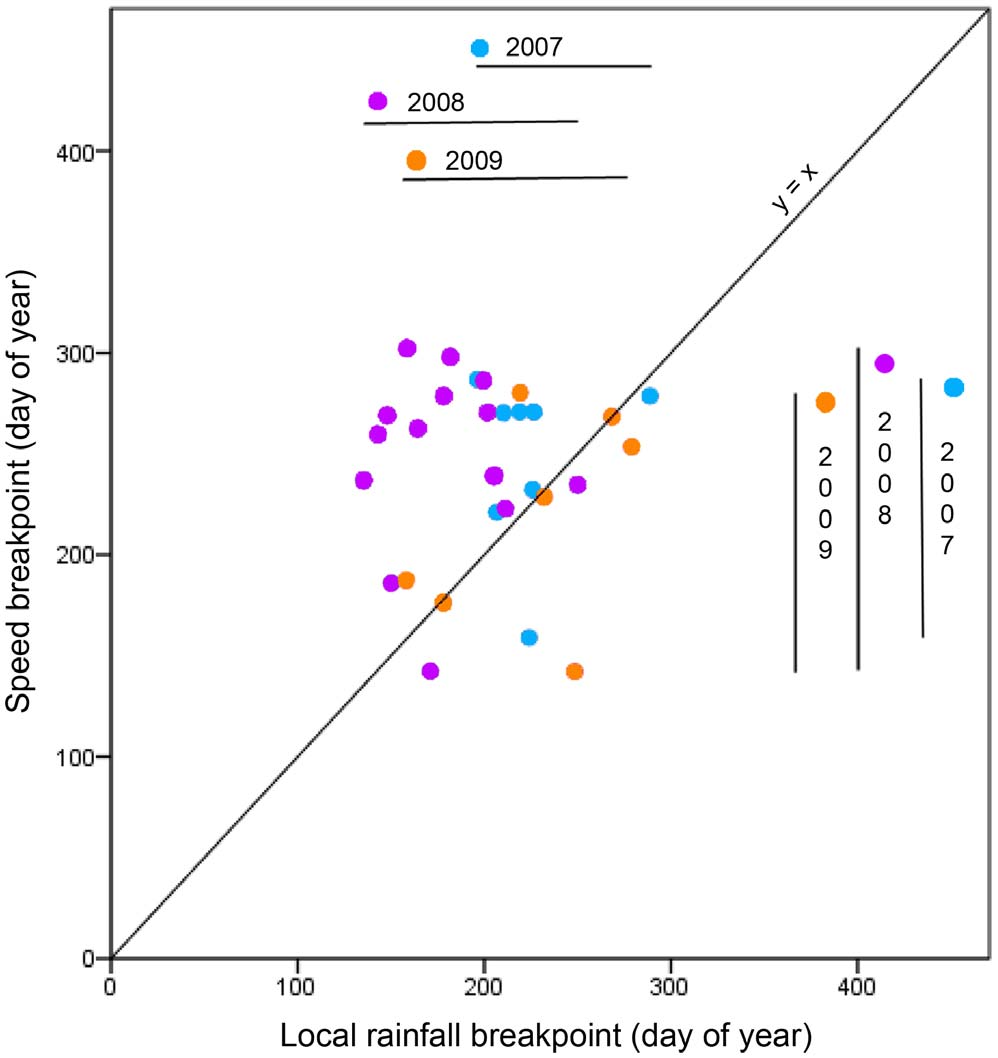
The effect of local rainfall on the average speed of elephant during the dry to wet season transition in Kruger National Park. Lower speed breakpoints are plotted against lower rainfall breakpoints for 8 collars in 2007 (blue), 14 collars in 2008 (purple) and 7 collars in 2009 (orange). Points above the line (y = x) represent speed breakpoints that occur after rainfall breakpoints; points below the line (y = x) represent speed breakpoints that occur before rainfall breakpoints. Rainfall and speed breakpoints that fall exactly on the line are equal to each other i.e. the breakpoints occur simultaneously. Vertical lines for each year represent the range in speed breakpoints (from Day 159 to 287 in 2007; Day 142 to 302 in 2008 and Day 142 to 280 in 2009). Horizontal lines represent the range in local rainfall (from Day 196 to 289 in 2007; Day 135 to 250 in 2008 and Day 158 to 279 in 2009).

We found that, overall, 35% of elephant increased their speed before the rainfall breakpoint, while 65% increased their speed after this breakpoint. When viewed separately, upper and lower breakpoints showed different trends: Lower breakpoints (dry to wet season transition): 29% of elephant increased their speed before lower rainfall breakpoints, 71% after (see Fig. 3); Upper breakpoints (wet to dry season transition): 63% of elephant increased their speed before upper rainfall breakpoints, 37% after. In general, values for mean speed, and the variance in mean speed, were at their highest during summer months when rainfall reached a peak; and at their lowest during the driest winter months.

Bivariate correlations revealed a strong positive relationship between speed, and both local (Pearson Correlation: r = 0.973; P<0.01) and regional (r = 0.982; P<0.01) rainfall breakpoints. However, when we used ‘year’ and ‘cluster’ as controls in a partial regression analysis, we found slightly lower, but still significant correlations: speed and local rainfall breakpoints; r = 0.752, P<0.01 and speed and regional rainfall breakpoints; r = 0.828, P<0.01. For upper breakpoints (wet to dry season transition), we found very little effect of either ‘year’ or ‘cluster’: speed and local rainfall: r = 0.913; speed and regional rainfall: r = 0.972. However, for lower breakpoints (dry to wet season transition), ‘year’ had a confounding effect on speed and rainfall breakpoints (speed and local rainfall: r = 0.04; P = 0.421; speed and regional rainfall: r = −0.003; P = 0.495). ‘Cluster’, as a second control, did not appear to affect the breakpoints (speed and local rainfall: r = 0.973; speed and regional rainfall: r = 0.981).

## Discussion

It is widely acknowledged that, within savanna environments, elephant movements are affected by seasonal changes in rainfall [11], [24], [38]. However, no prior studies have used these movement patterns to discern temporal breakpoints or shifts in behaviour across seasons. By examining variation in elephant speed across broad scales (multiple years), we have allowed the behaviour of individual elephant to reveal distinct seasonal shifts, rather than prescribing arbitrarily defined seasons. In this way, the timing of our seasons is biologically relevant to the species, and does not depend on coarse-scale measures of an external variable which may not reflect the true variation in the species’ behaviour. Examination of elephant movement patterns in conjunction with rainfall patterns indicated that all herds changed their behaviour at two distinct thresholds: at the end of the dry season before the first rains commenced, and at the end of the wet season, during the period of the highest average daily rainfall. These changes coincided with the major rainfall breakpoints, which signal a seasonal transition between wet and dry, and dry and wet periods.

In general, elephant in KNP increased their speed during the wet season (summer months) up until a maximum threshold point at the onset of the dry season, following which their speed reduced, until a minimum threshold point at the end of the dry season. Beyond this point, at the onset of the wet season, their speed increased once more (see Fig. 2). The lower breakpoints indicate that a change from dry to wet season conditions triggered a release from constrained movement behaviour, which is consistent with the ‘dry season bottleneck’ theory proposed for herbivores in environments where there are markedly different seasons [12]. Herbivores are restricted during dry periods, since both forage quality and quantity are reduced; however, there is a release from these constraints once the wet season commences [12]. Female elephant in KNP are most restricted in the dimension associated with rainfall and herbaceous biomass [14]. During the dry season, females, and in particular weaned calves, are susceptible to stress as a result of the decreased nutritional value of forage [39]. Thus, in order to conserve energy, female elephant in KNP are likely to restrict their movement at drier times of the year when forage quantity and quality is lower. The decrease in speed and lower variance in speed during the dry season also suggests that elephant in KNP may be using smaller areas more intensively. Since past research has revealed that elephant use riparian vegetation and low-lying thickets during the dry season [40]–[42], these habitats with available browse may be more intensively used, and are thus vulnerable to higher levels of impact.

There is a strong inter-annual variability in rainfall patterns in savannas, where cycles of above- and below-average rainfall occur in different years [43]. This variability is likely to affect the timing of the rainfall breakpoints in different years, and thus confound the relationship between elephant speed and rainfall. Between different years, the relationship between rainfall and speed breakpoints in the dry-wet period (lower breakpoints) appears to be highly variable: differences between average speed and local rainfall breakpoints range from 24 days in 2007, to 71 days in 2008 and 7 days in 2009 (Appendix S2). Monthly rainfall data from KNP indicate that in 2008, conditions were drier, with below-average rainfall measurements at the majority of stations for eight of the 12 months (SANParks unpublished data). This may explain the differences in the timing of speed and rainfall breakpoints during this year.

Rainfall directly affects primary productivity in savanna systems [44]. In north-western and south-eastern regions in South Africa, both forage quality and abundance within savannas is positively correlated with rainfall, and forage quality gradually increases after mid-August [22]. Since the lower breakpoints (dry to wet season transition) in elephant speed (in 2007, 2008 and 2009) occurred from early August up until early September (Appendix S2), it is likely that this increase in movement was a response by elephant to increased forage quality. The appearance of leaves in certain savanna tree species have been shown to precede rainfall events, (e.g. [45], [46]), an occurrence which is likely to be caused by changes in day length [47]. Thus, female elephant and calves may rely on early flushes in certain tree species prior to rainfall events [48]. In this study, female elephant increased their movements at the end of the dry season, which indicates that they may have tracked these early flushes and moved into new habitats or areas where browse was available. The reason that elephant decreased their speed during the wet-dry season threshold, during a period of the highest average daily rainfall, is less clear. Elephant in Kruger demonstrate a switch from a 50% inclusion of grass in their diet during the wet months, to a 10% inclusion during the dry months [49]. Thus, since areas of palatable grass are more widely spread during the early-to-mid wet season [43], [49], we could hypothesise that elephant moved more during this period (i.e. increased their speed) to access these areas. Later in the season, certain areas become less palatable (e.g. sourveld) [43], resulting in reduced availability of graze and slower movements by elephant. An investigation of elephant habitat-use over this period would be necessary to verify these hypotheses.

Although abiotic factors such as rainfall, snowfall or temperature may be informative when examined alongside movement patterns (e.g. [2], [9]), in isolation, they may bias the estimation of time frames which become arbitrary to the species in question. Thus it is necessary to identify biologically relevant breakpoints over time, in order to assess changes in behaviour relating to the spatial use of habitats by elephant and other large herbivores in different environments. This in turn allows for a better understanding of animal responses to seasonally available resources, and variable environmental conditions [13]. In terrestrial systems where large herbivores play a vital role as ecosystem drivers [50]–[52], it is important to elucidate not only where individuals are spatially distributed, but when behavioural changes occur and which factors play a key role in determining these shifts. This allows for the implementation of more effective management protocols for threatened species and habitats.

This study provides a unique method for identifying appropriate temporal breakpoints over broad scales, which can be applied to other species, in particular large mammals capable of carrying GPS collars. Within this framework, variables that may influence the movement of an animal (e.g. rainfall, snowfall, temperature) can be examined alongside movement patterns, in order to examine potential interactions. Although we have used a broad-scale approach, other temporal scales (yearly, monthly, weekly), or a multi-scale approach may be applied. Thus the methods used in this study provide useful tools that can be used to extract a comprehensive description of the animal’s movement behaviour, in association with important variables.

## Supporting Information

Appendix S1 **Table 1 Results for speed, local rainfall and regional rainfall breakpoints from all collars, obtained using multi-year piecewise regression models.**
(DOC)

Appendix S2 **Table 1 Average speed, local rainfall and regional rainfall breakpoints for all collars, obtained using multiyear piecewise regression models.**
(DOC)
