# Supplementary material for: Animal Perception of Seasonal Thresholds: Changes in Elephant Movement in Relation to Rainfall Patterns
Source: PLoS One. 2012 Jun 27;7(6):e38363. doi: 10.1371/journal.pone.0038363 (PMC3384670; doi:10.1371/journal.pone.0038363)
Supplement: Appendix S1 — Table 1 Results for speed, local rainfall and regional rainfall breakpoints from all collars, obtained using multi-year piecewise regression models. (DOC) [file pone.0038363.s001.doc]

**Appendix S1**

| **Collars** | **BP #** | **Speed BP (multiyear)** | **l/u BP** | **Speed BP (year)** | **year** | **Loc R** | **Loc R (year)** | **year** | **Reg R BP**  **(multiyear)** | **Reg R (year)** | **year** |
| --- | --- | --- | --- | --- | --- | --- | --- | --- | --- | --- | --- |
| AM105 | 1 | 286.80 | l | 286.80 | 2007 | 196.27 | 196.27 | 2007 | 223.04 | 223.04 | 2007 |
| AM105 | 2 | 312.11 | u | 312.11 | 2007 | 358.85 | 358.85 | 2007 | 336.18 | 336.18 | 2007 |
| AM105 | 3 | 507.28 | l | 142.28 | 2008 | 536.01 | 171.01 | 2008 | 563.04 | 198.04 | 2008 |
| AM106 | 1 | 232.20 | l | 232.20 | 2007 | 225.96 | 225.96 | 2007 | 222.96 | 222.96 | 2007 |
| AM106 | 2 | 323.42 | u | 323.42 | 2007 | 355.93 | 355.93 | 2007 | 336.15 | 336.15 | 2007 |
| AM106 | 3 | 601.90 | l | 236.90 | 2008 | 500.35 | 135.35 | 2008 | 560.48 | 195.48 | 2008 |
| AM106 | 4 | 756.48 | u | 26.48 | 2009 | 773.76 | 43.76 | 2009 | 741.15 | 11.15 | 2009 |
| AM106 | 5 | 872.15 | l | 142.15 | 2009 | 978.43 | 248.43 | 2009 | 990.16 | 260.16 | 2009 |
| AM107 | 1 | 158.93 | l | 158.93 | 2007 | 224.02 | 224.02 | 2007 | 208.16 | 208.16 | 2007 |
| AM107 | 2 | 346.84 | u | 346.84 | 2007 | 341.50 | 341.50 | 2007 | 350.65 | 350.65 | 2007 |
| AM107 | 3 | 587.83 | l | 222.83 | 2008 | 576.26 | 211.26 | 2008 | 546.66 | 181.66 | 2008 |
| AM108 | 1 | 220.96 | l | 220.96 | 2007 | 206.64 | 206.64 | 2007 | 192.99 | 192.99 | 2007 |
| AM108 | 2 | 387.74 | u | 22.74 | 2008 | 368.44 | 3.44 | 2008 | 356.84 | 356.84 | 2007 |
| AM108 | 4 | 551.06 | l | 186.06 | 2008 | 515.26 | 150.26 | 2008 | 558.67 | 193.67 | 2008 |
| AM110 | 1 | 270.72 | l | 270.72 | 2007 | 226.39 | 226.39 | 2007 | 220.61 | 220.61 | 2007 |
| AM110 | 2 | 324.52 | u | 324.52 | 2007 | 348.73 | 348.73 | 2007 | 335.02 | 335.02 | 2007 |
| AM110 | 3 | 667.24 | l | 302.24 | 2008 | 523.70 | 158.70 | 2008 | 551.71 | 186.71 | 2008 |
| AM110 | 4 | 744.98 | u | 14.98 | 2009 | 762.77 | 32.77 | 2009 | 743.26 | 13.26 | 2009 |
| AM110 | 5 | 983.52 | l | 253.52 | 2009 | 1008.90 | 278.90 | 2009 | 987.74 | 257.74 | 2009 |
| AM91 | 1 | 270.75 | l | 270.75 | 2007 | 219.01 | 219.01 | 2007 | 188.13 | 188.13 | 2007 |
| AM91 | 2 | 311.27 | u | 311.27 | 2007 | 363.81 | 363.81 | 2007 | 357.11 | 357.11 | 2007 |
| AM91 | 3 | 634.18 | l | 269.18 | 2008 | 513.01 | 148.01 | 2008 | 554.85 | 189.85 | 2008 |
| AM91 | 4 | 752.03 | u | 22.03 | 2009 | 746.29 | 16.29 | 2009 | 742.67 | 12.67 | 2009 |
| AM91 | 5 | 998.45 | l | 268.45 | 2009 | 998.32 | 268.32 | 2009 | 991.24 | 261.24 | 2009 |
| AM93 | 1 | 270.19 | l | 270.19 | 2007 | 210.24 | 210.24 | 2007 | 229.38 | 229.38 | 2007 |
| AM93 | 2 | 312.34 | u | 312.34 | 2007 | 361.34 | 361.34 | 2007 | 324.57 | 324.57 | 2007 |
| AM93 | 3 | 643.75 | l | 278.75 | 2008 | 543.31 | 178.31 | 2008 | 556.32 | 191.32 | 2008 |
| AM93 | 4 | 746.73 | u | 16.73 | 2009 | 741.57 | 11.57 | 2009 | 735.28 | 5.28 | 2009 |
| AM99 | 1 | 278.66 | l | 278.66 | 2007 | 288.77 | 288.77 | 2007 | 290.14 | 290.14 | 2007 |
| AM99 | 2 | 306.10 | u | 306.10 | 2007 | 274.09 | 274.09 | 2007 | 273.82 | 273.82 | 2007 |
| AM99 | 5 | 599.63 | l | 234.63 | 2008 | 615.10 | 250.10 | 2008 | 589.41 | 224.41 | 2008 |
| AM239 | 1 | 624.61 | l | 259.61 | 2008 | 508.00 | 143.00 | 2008 | 556.64 | 191.64 | 2008 |
| AM239 | 2 | 717.04 | u | 352.04 | 2008 | 785.32 | 55.32 | 2009 | 742.92 | 12.92 | 2009 |
| AM239 | 3 | 917.30 | l | 187.30 | 2009 | 887.96 | 157.96 | 2009 | 931.91 | 201.91 | 2009 |
| AM253 | 1 | 604.10 | l | 239.10 | 2008 | 570.20 | 205.20 | 2008 | 570.41 | 205.41 | 2008 |
| AM254 | 1 | 651.43 | l | 286.43 | 2008 | 564.23 | 199.23 | 2008 | 560.44 | 195.44 | 2008 |
| AM306 | 1 | 635.56 | l | 270.56 | 2008 | 566.74 | 201.74 | 2008 | 561.43 | 196.43 | 2008 |
| AM306 | 2 | 717.83 | u | 352.83 | 2008 | 696.14 | 331.14 | 2008 | 742.91 | 377.91 | 2009 |
| AM306 | 3 | 1010.22 | l | 280.22 | 2009 | 949.20 | 219.20 | 2009 | 932.69 | 202.69 | 2009 |
| AM307 | 1 | 663.11 | l | 298.11 | 2008 | 546.79 | 181.79 | 2008 | 562.75 | 197.75 | 2008 |
| AM307 | 2 | 748.05 | u | 18.05 | 2009 | 726.37 | 361.37 | 2008 | 741.59 | 11.59 | 2009 |
| AM307 | 3 | 958.58 | l | 228.58 | 2009 | 962.09 | 232.09 | 2009 | 969.74 | 239.74 | 2009 |
| AM308 | 1 | 627.45 | l | 262.45 | 2008 | 529.32 | 164.32 | 2008 | 562.83 | 197.83 | 2008 |
| AM308 | 2 | 760.55 | u | 30.55 | 2009 | 775.50 | 45.50 | 2009 | 743.15 | 13.15 | 2009 |
| AM308 | 3 | 906.32 | l | 176.32 | 2009 | 907.94 | 177.94 | 2009 | 931.67 | 201.67 | 2009 |

BP = Breakpoint BP (year) = breakpoint in days of a single year

Loc R = Local Rainfall BP (multiyear) = breakpoint over cumulative days (3 year period)

Reg R = Regional Rainfall

l/u BP = lower/upper Breakpoint

BP # = Breakpoint number
