# Supplementary material for: Animal Perception of Seasonal Thresholds: Changes in Elephant Movement in Relation to Rainfall Patterns
Source: PLoS One. 2012 Jun 27;7(6):e38363. doi: 10.1371/journal.pone.0038363 (PMC3384670; doi:10.1371/journal.pone.0038363)
Supplement: Appendix S2 — Table 1 Average speed, local rainfall and regional rainfall breakpoints for all collars, obtained using multiyear piecewise regression models. (DOC) [file pone.0038363.s002.doc]

**Appendix S2**

| **Lower BP's** |  | **BP calc** | **Spd BP (multiyear)** | **BP (year)** | **Loc R BP (multiyear)** | **BP (year)** | **Reg R BP (multiyear)** | **BP (year)** |
| --- | --- | --- | --- | --- | --- | --- | --- | --- |
|  | **2007** | **Avg** | 248.65 | 248.65 | 224.66 | 224.66 | 221.93 | 221.93 |
|  |  | **CI** | 127.87 | 127.87 | 92.51 | 92.51 | 102.01 | 102.01 |
|  |  | **date** | 06-Sep |  | 13-Aug |  | 10-Aug |  |
|  |  | **diff spd & loc R** | 23.99 |  |  |  |  |  |
|  |  | **diff spd & reg R** | 26.73 |  |  |  |  |  |
|  | **2008** | **Avg** | 614.22 | 249.22 | 543.45 | 178.45 | 561.12 | 196.12 |
|  |  | **CI** | 159.95 | 159.95 | 114.75 | 114.75 | 42.75 | 42.75 |
|  |  | **date** | 05-Sep |  | 26-Jun |  | 14-Jul |  |
|  |  | **diff spd & loc R** | 70.78 |  |  |  |  |  |
|  |  | **diff spd & reg R** | 53.11 |  |  |  |  |  |
|  | **2009** | **Avg** | 949.51 | 219.51 | 956.12 | 226.12 | 962.16 | 232.16 |
|  |  | **CI** | 138.07 | 138.07 | 120.94 | 120.94 | 59.57 | 59.57 |
|  |  | **date** | 07-Aug |  | 13-Aug |  | 19-Aug |  |
|  |  | **diff spd & loc R** | 6.61 |  |  |  |  |  |
|  |  | **diff spd & reg R** | 12.66 |  |  |  |  |  |
| **Upper BP's** |  | **BP calc** | **Spd BP (multiyear)** | **BP (year)** | **Loc R BP (multiyear)** | **BP (year)** | **Reg R BP (multiyear)** | **BP (year)** |
|  | **2007** | **Avg** | 319.51 | 319.51 | 343.46 | 343.46 | 330.50 | 330.50 |
|  |  | **CI** | 40.75 | 40.75 | 89.71 | 89.71 | 83.28 | 83.28 |
|  |  | **date** | 16-Nov |  | 09-Dec |  | 26-Nov |  |
|  |  | **diff spd & loc R** | 23.95 |  |  |  |  |  |
|  |  | **diff spd & reg R** | 10.99 |  |  |  |  |  |
|  | **2008** | **Avg** | 717.43 | 352.43 | 696.14 | 331.14 | / | / |
|  |  | **CI** | 0.79 | 0.79 | 0.00 | 0.00 | / | / |
|  |  | **date** | 17-Dec |  | 26-Nov |  |  |  |
|  |  | **diff spd & loc R** | 21.29 |  |  |  |  |  |
|  |  | **diff spd & reg R** | 0.00 |  |  |  |  |  |
|  | **2009** | **Avg** | 753.55 | 23.55 | 764.20 | 34.20 | 741.62 | 11.62 |
|  |  | **CI** | 15.57 | 15.57 | 43.75 | 43.75 | 7.98 | 7.98 |
|  |  | **date** | 23-Jan |  | 02-Feb |  | 11-Jan |  |
|  |  | **diff spd & loc R** | 10.65 |  |  |  |  |  |
|  |  | **diff spd & reg R** | 11.94 |  |  |  |  |  |

BP = Breakpoint R = Rainfall

Spd = Speed Diff = difference

Loc = local Avg = Average

CI = 95% Confidence Interval Reg = Regional

BP (year) = breakpoint in days of a single year

BP (multiyear) = breakpoint over cumulative years (3 year period)
